# Unveiling Familial Hypercholesterolemia—Review, Cardiovascular Complications, Lipid-Lowering Treatment and Its Efficacy

**DOI:** 10.3390/ijms25031637

**Published:** 2024-01-29

**Authors:** Piotr Fularski, Joanna Hajdys, Gabriela Majchrowicz, Magdalena Stabrawa, Ewelina Młynarska, Jacek Rysz, Beata Franczyk

**Affiliations:** 1 Department of Nephrocardiology, Medical University of Lodz, ul. Zeromskiego 113, 90-549 Lodz, Poland; 2 Department of Nephrology, Hypertension and Family Medicine, Medical University of Lodz, ul. Zeromskiego 113, 90-549 Lodz, Poland

**Keywords:** familial hypercholesterolemia, atherosclerosis, cardiovascular disease, LDL-C

## Abstract

Familial hypercholesterolemia (FH) is a genetic disorder primarily transmitted in an autosomal-dominant manner. We distinguish two main forms of FH, which differ in the severity of the disease, namely homozygous familial hypercholesterolemia (HoFH) and heterozygous familial hypercholesterolemia (HeFH). The characteristic feature of this disease is a high concentration of low-density lipoprotein cholesterol (LDL-C) in the blood. However, the level may significantly vary between the two mentioned types of FH, and it is decidedly higher in HoFH. A chronically elevated concentration of LDL-C in the plasma leads to the occurrence of certain abnormalities, such as xanthomas in the tendons and skin, as well as corneal arcus. Nevertheless, a significantly more severe phenomenon is leading to the premature onset of cardiovascular disease (CVD) and its clinical implications, such as cardiac events, stroke or vascular dementia, even at a relatively young age. Due to the danger posed by this medical condition, we have investigated how both non-pharmacological and selected pharmacological treatment impact the course of FH, thereby reducing or postponing the risk of clinical manifestations of CVD. The primary objective of this review is to provide a comprehensive summary of the current understanding of FH, the effectiveness of lipid-lowering therapy in FH and to explain the anatomopathological correlation between FH and premature CVD development, with its complications.

## 1. Introduction

FH is one of the most prevalent hereditary disorders in the world and is associated with high levels of circulating LDL-C [1]. As such, it predisposes patients to the formation of extravascular deposits, forming xanthomas of the skin and tendons (mainly the Achilles tendon, finger extensor tendons, and also the patellar and triceps tendons), corneal arcus, xanthelasma and vascular deposits, resulting in rapidly progressive atherosclerosis and coronary heart disease (CHD) at a young age [2,3]. Consequently, these patients are characterized by increased morbidity and mortality [2].

FH occurs in the heterozygous type at a frequency of 1 in 250–300 cases, while the homozygous type occurs at a frequency of 1 in 250,000–360,000 cases [4,5,6]. In spite of its prevalence, FH remains largely underdiagnosed and therefore undertreated [2]. If left untreated, patients develop coronary artery disease (CAD) earlier than healthy individuals, occurring earlier than the age of 55 in men and 60 in women. Nevertheless, with prompt diagnosis and adequate treatment, the risk of developing CAD can be significantly reduced [7].

The diagnosis of FH is mainly established on the basis of the clinical presentation, although diagnostic criteria can also be used. The most commonly applied diagnostic criteria for HeFH are the Dutch Lipid Clinic Network diagnostic criteria (Table 1), although these cannot be used in children [8,9]. For children as well as adults, the Simon Broome criteria can be applied, which include physical examination findings, serum cholesterol levels, DNA testing and family history [10]. For MEDPED, it is also necessary to know the LDL-C levels of the other members of the family [11]. For the diagnosis of HoFH, the criteria updated in Table 2 [4] are used.

In the diagnosis of FH, it is crucial to measure the fasting LDL-C at least twice and to exclude possible secondary causes such as hypothyroidism [12].

To verify the diagnosis, mutations in pathogenic genes can be identified. Nonetheless, the detection rate of the relevant mutations in clinically definite or probable FH patients is uncertain, suggesting either the presence of a polygenic cause of FH or the possibility of the involvement of genes not yet identified [7].

When FH is diagnosed in a patient, cascade screening should be performed in the remaining family members by means of TC- or LDL-C-level analysis. Diagnostic genetic testing can be performed for known mutations [13,14].

### 1.1. Monogenic FH

FH is predominantly inherited as an autosomal dominant trait, except in regions where there is a high degree of consanguinity. In this case, it may be inherited as an autosomal recessive trait [15,16]. Nonetheless, the phenotype of FH may vary considerably in terms of the lipid profile and clinical manifestations, and it may respond differently to pharmacological treatment due to the large diversity of possible mutations causing FH and the additional potential involvement of other genes [17].

#### 1.1.1. Autosomal Dominant Hypercholesterolemia

Autosomal dominant hypercholesterolemia (ADH) can result from loss-of-function (LOF) mutations in either the *LDLR* or *APOB* genes, or from gain-of-function (GOF) mutations in the *PCSK9* gene, but it can also be caused by the contribution of certain FH variants in the *APOE* gene [18]. Within the *LDLR* gene, over 1000 different mutations have been identified that may contribute to the development of FH [2], and approximately 95% of cases occur as a result of a mutation in the *LDLR* gene. Mutations have the potential to cause reduced or complete loss of gene function and are therefore associated with a more severe course of the disease [17]. In comparison, patients with mutations in the *APOB* or *PCSK9* genes tend to have a milder disease phenotype [17].

The LDLR Gene

The low-density lipoprotein receptor (LDLR) gene encodes a transmembrane receptor located on the surface of cells that induces LDL transport into cells via endocytosis [3,19]. Over the years, thousands of *LDLR* gene variants triggering FH have been recorded in historical and general databases [20]. They are currently divided into pathogenic, probably pathogenic and variant of unknown significance (VUS) [21,22].

The most common molecular changes in the *LDLR* gene are small nucleotide variations. These occur throughout the gene. Nucleotide changes that result in a partial loss of or change in receptor function are termed defective alleles, whereas variants that result in a completely abnormal or absent protein result in a complete loss of LDL receptor function and are termed null (or negative) alleles [23,24,25]. Patients with these variants have a more severely affected phenotype, defined not only by significantly elevated LDL-C but also by a higher incidence of tendon xanthomas, carotid atherosclerosis or CHD [19,26]. Although there is no specific mutation site, studies show that mutations in exon 4 are correlated with a severe FH phenotype. This is likely due to the encoding of three of the seven repeats of the region required for LDL binding by apo B [19,26]. The occurrence of common pathogenic variants is associated with certain ethnic or geographical groups due to the founder effect [27]. The initial mutations that were identified in the LDLR gene are copy number alterations and account for approximately 10% of the causative variants in FH [28]. In addition, with advances in technology and the accessibility of whole genome sequencing (WGS), new deep intron variants in the *LDLR* gene have been detected in patients with FH and have been noted to segregate well in families with the FH phenotype [29,30]. The *LDLR* intron regions should be tested in mutation-negative FH patients, as some intron variants may cause premature stop codon appearance [29,30].

A reduced amount of receptors on the cell surface or the presence of dysfunctional receptors is the consequence of pathogenic variants in the *LDLR* gene. They have been classified into five classes according to their influence on LDLR production (Table 3) [31,32].

The main genetic feature of FH is its familial occurrence. Extremely uncommon are the pathogenic de novo variants within the *LDLR* gene. Therefore, identification of FH at the molecular level should lead us in the direction of screening relatives, since the risk of having a mutation is 50% [33,34].

##### The APOB Gene

Apo B, as one of the LDLr ligands, is encoded by the apolipoprotein B (*APOB*) gene. The variants that have been reported to the central databases are mainly LOF variants, provoking hypobetalipoproteinemia. However, it has been observed that some missense variants may also cause ADH [35,36]. These are notably variations in the critical region of exon 26 of the *APOB* gene [37,38]. Variations in this region cause defective LDL receptor binding by reducing the affinity of apo B for LDLr [39,40].

WGS is currently finding new ADH-inducing variants in known genes. Some variants in the *APOB* gene can increase LDL-C in the blood and are responsible for its defective uptake by the liver [41]. Furthermore, they reduce the ability of apo B to bind to the LDLr receptor [42]. Some studies have shown that variations of the *APOB* gene lead to as much as a 40% decrease in internalization in both lymphocytes and HepG2 cells [35,37].

It has been observed that mutations occurring in the *APOB* gene may undergo incomplete penetrance in families [35]. Therefore, testing of the entire *APOB* gene is increasingly recommended due to the possibility that more ADH-inducing variants may exist beyond the conventionally tested regions [35,37].

##### The PCSK9 Gene

A protein that inhibits the uptake of LDL-C from the bloodstream is encoded by the proprotein convertase subtilisin/kexin type 9 (*PCSK9*) gene. The protein is released into the circulation and attaches itself to the LDLr receptor [43]. The PCSK9–LDLr complex is then directed to the lysosome, where it undergoes lysosomal degradation and thus reduces the recycling of LDLr to the cell surface [43,44]. Variants within PCSK9 can be either GOF or LOF.

Patients with the GOF variant, which occurs in approximately 1% of FH cases, have fewer receptors on the cell surface for LDL, particularly on hepatocytes [1]. Thus, there is an excessive accumulation of LDL-C in the blood in these individuals due to the lack of clearance [43]. In mature PCSK9 proteins, GOF variants may have different effects. Variants may result in reduced cleavage and subsequent secretion of PCSK9, enhanced half-life or stability as a result of reduced cleavage and complete or partial deactivation by furin [45], and increased affinity to bind to the LDLr receptor as compared to the normal form of PCSK9, resulting in lysosomal degradation [46].

LOF variants of the *PCSK9* gene are associated with lower mean LDL-C levels and a reduced risk of CHD [47,48].

Pathogenic and probably pathogenic variants of the *PCSK9* gene are approximately 36 in the genomic database and are of little relevance to the molecular diagnosis of FH compared to *LDLR* or *APOB* genes [22,49].

##### The APOE Gene

The *APOE* gene is responsible for the formation of apolipoprotein E (apo E), which is a major component of lipoprotein metabolism. Variations within this gene lead to variable LDL-C values and cause various dyslipidemias [50,51]. Certain variants of the *APOE* gene have recently been linked to ADH. Variants have been identified in families with FH without mutations in another FH-related gene, such as *LDLR*, *APOB*, or *PCSK9* [18,52]. Therefore, the *APOE* gene is also considered to be associated with the occurrence of FH, while contributing little to the molecular pathology of FH.

#### 1.1.2. Autosomal Recessive Hypercholesterolemia

FH can also be inherited in an autosomal recessive (ARH) manner. HoFH is an unusual and life-threatening disorder that manifests as extensive jaundice, premature and progressive CVD and TC levels above 13 mmol/L (>500 mg/dL) [1,7]. Nevertheless, according to an update from the European Atherosclerosis Society, a currently untreated LDL-C level >10 mmol/L (>∼400 mg/dL) should suggest the presence of HoFH and needs to be further assessed [4]. The majority of these patients progress to CAD and aortic stenosis by the age of 20, resulting in rapid death by 30 years of age [53]. Frequently, patients with HoFH who carry two receptor mutations suffer myocardial infarction well before 10 years of age [53,54]. It is therefore crucial to identify children quickly and refer them to a specialist clinic to initiate intensive LDL-lowering drug therapy [7].

With the ability to sequence regional genes that are expressed in the liver and the simultaneous use of linkage analysis with homozygosity mapping, the gene responsible for ARH has been identified and designated *LDLRAP1* [55]. The LDL receptor adaptor protein1 (*LDLRAP1*) gene encodes an adaptor protein that binds to cell surface receptors, including LDLr, through its structure [56]. By interacting with clathrin, it is involved in the endocytosis of LDLr. The adaptor protein is therefore an essential component of the specific LDL-LDLr endocytic pathway [57,58]. So far, 15 pathogenic variants of the *LDLRAP1* gene have been detected in which nucleotide insertions or deletions occur, which are associated with a shift in the reading frame and truncation of the protein [59].

**Figure 1:**
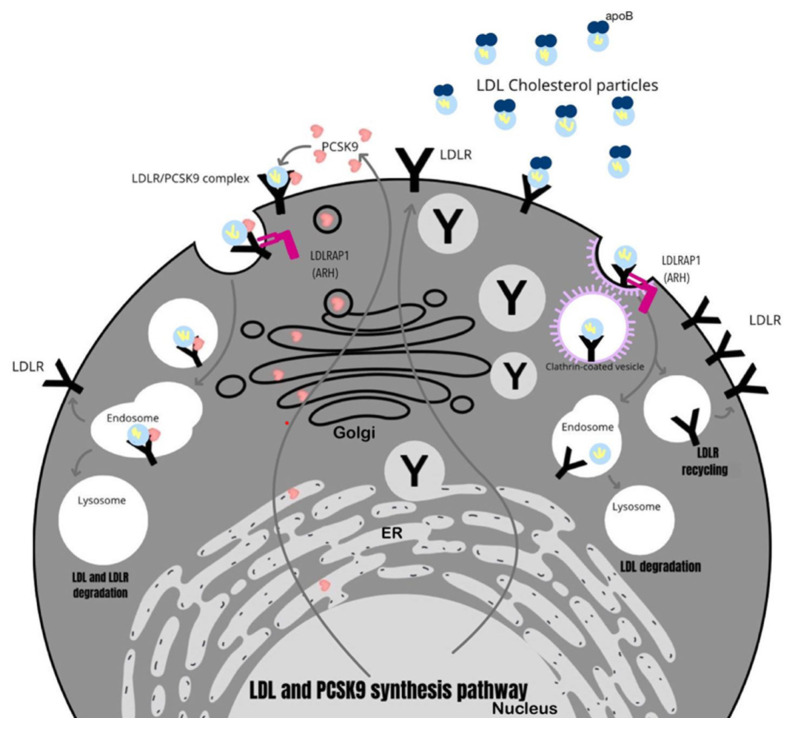
The role of key genes involved in the development of FH.

The functions performed by the most relevant genes involved in the development of FH are shown in Figure 1.

#### 1.1.3. Complex and Double Heterozygosity

It is occasionally the case that patients with FH have more than one variant in their FH-related genes. This situation has been termed double or compound heterozygosity. Compound heterozygosity occurs when the two alleles of a gene are two separate pathogenic variants, while double heterozygosity occurs when the patient has pathogenic variants in two different genes, namely *LDLR* and *APOB, LDLR* and *PCSK9*, *LDLR* and *LDLRAP1*, or *APOB* and *PCSK9* [60,61,62,63].

The occurrence of heterozygous FH variants may produce differential effects. Regarding which gene variants are present, patients may have an equal or possibly worse phenotype than those with HoFH, which is correlated with a more severe presentation and earlier onset of cardiovascular complications [64]. Likewise, cases have been observed where there is a variant that lowers the LDL-C levels, leading to a phenotype that may be mild or even normal. This may be the result of compensation between a pathogenically mutated LDLr variant and a variant with increased recycling of functional LDLr [65].

### 1.2. Polygenic FH

Studies highlight the genetic complexity underlying common dyslipidemias, including hypercholesterolemia. A meta-analysis of genome-wide association studies (GWAS) identified as many as 941 genomic loci relevant to lipids [66,67]. Up to now, the detection of a pathogenic variant has been successful in 60–80% of patients with definite FH as defined by the diagnostic criteria of the Dutch Lipid Clinic Network, but only 20–30% in patients with possible FH [68,69,70]. It was concluded from this that the disorder may be a result of the cumulative effect of common alleles that together cause elevated LDL-C levels but individually have a minor effect [68].

Thus, the polygenic risk of the 12 most frequent alleles that elevate the blood LDL-C levels was estimated in individuals with FH who were clinically diagnosed with FH with or without an identified FH-causing variant [71]. It appeared that those without an identified variant had higher mutation scores than those with an identified variant, explaining a potential polygenic effect on severe hypercholesterolemia among patients with FH whose variant was not identified [71].

The existence of disease variability, expressed by variations in LDL-C levels, as well as cardiovascular complications, may also be considered against the polygenic background [2]. Variability is observed among families but also among members of the same family. This may be due to genetic heterogeneity involving various genes responsible for the disease onset but also to different pathogenic variants in the same disease gene [72]. However, this explains the variation between families. In the case of variability within family members, an explanation may be the hypothesis of an additional polygenic effect on disease expression, which may exacerbate or attenuate the effect of the mutation causing FH [2].

Over the years since the use of large-scale WES and whole genes sequencing (WGS) studies, further variants have been identified that are potentially responsible for the occurrence of FH [73]. With the observation of patients with variants present, it appears that not all have a deleterious effect and that they are not always related to the blood cholesterol levels [68]. This could be attributed to multigene causes or the presence of as yet unidentified variants. However, this does not change the fact that many families still remain without knowledge of the reason for their FH [2,7].

## 2. Correlation between FH and Atherosclerotic Cardiovascular Disease

In patients suffering from FH, specific mutations most frequently occur in the sequences of genes responsible for encoding the LDLR, APOB or PCSK9. These mutations are leading to insufficient LDL-C clearance, which implies an elevated level of the mentioned lipoproteins in the bloodstream [74]. This is particularly significant, because the raised level of LDL-C is the dominant factor in the onset and the further advancement of atherosclerosis [75].

The likelihood of atherosclerosis development varies significantly in different locations within the vascular system. Especially susceptible areas are those with bifurcations or curvatures that cause disturbances in the blood flow [76]. Disruptions in blood circulation in such places result in increased local stress, subsequently weakening the connections between endothelial cells, which consequently facilitates the passage of LDL-C through the endothelial barrier and promotes the deposition of low-density lipoprotein cholesterol molecules within the subendothelial space [77]. The accumulation of LDL-C is specifically intensified in patients suffering from FH, mainly due to the fact that hypercholesterolemia itself is a factor enhancing the transmission of LDL-C into the wall of the artery [78]. Moreover, if any section of the endothelium becomes damaged, it initiates the secretion of molecules whose task is to attract monocytes. Afterwards, monocytes migrate from the bloodstream into the tunica intima, where they transform into macrophages, as well as they begin to produce oxidizing substances that oxidize the LDL-C. Ox-LDL-C (oxidized low-density lipoprotein cholesterol) is then absorbed via macrophages, leading to their overload and the formation of foam cells [79]. Foam cells are also involved in maintaining and intensifying inflammation through the secretion of cytokines, additional attraction of monocytes, or by activating the vascular smooth muscle cells (VSMCs). What is more, they contribute to the enlargement of the necrotic core, which results in atherosclerotic plaque formation. With the passage of time, its dimensions increase, which leads to the vessel narrowing [80]. The atheromatous plaque is covered by a fibrous cap that consists of VSMCs, which serves to stabilize it, thus protecting it from rupturing for as long as it is able to resist the pressure from pulsating blood circulation [81]. Over time, the plaques begin to undergo the process of calcification, which in itself is an accurate indicator of atherosclerosis advancement. Although the degree of calcification is related to the size of the atherosclerotic plaques, it does not consistently correlate with its ability to rupture [82]. As atherosclerosis progresses, the necrotic area enlarges and the fibrous cap becomes thinner. This may further result in an interaction between the necrotic core and blood clotting factors, potentially provoking thrombosis [83]. Atherosclerosis undoubtedly constitutes the foundation of atherosclerotic cardiovascular disease (ASCVD), whereas development of atherosclerosis is strongly promoted by prolonged exposure to an elevated concentration of LDL-C in the blood, which typically occurs in FH [84,85]. ASCVD may manifest acutely in various ways, including cardiac event, stroke or transient ischemic attack. It may also be manifested chronically as, among others, stable angina pectoris, vascular dementia or peripheral arterial disease [86].

## 3. The Basis for Management of Familial Hypercholesterolemia

Familial hypercholesterolemia is strongly related to CVD, and data suggest that individuals with FH have a 10 times higher risk of CHD compared with the general population [1,17]. The cumulative LDL-C burden high enough to cause CHD is reached significantly earlier in FH patients (around 12.5 years) compared to patients without FH (around 55 years) [17].

Consequently, cholesterol-lowering therapies should be administered as soon as possible [7]. Moreover, the ESC/EAS highly suggest using imaging techniques to detect atherosclerosis in asymptomatic patients [7]. The ESC/EAS guidelines for the management of dyslipidemias from 2019, the ESC guidelines on cardiovascular disease prevention from 2021, and the multisociety guideline on the management of blood cholesterol from 2018 state that in most patients with FH, treatment with high-intensity statin therapy should be initiated combined with ezetimibe [7,87,88]. PCSK9 inhibitors are recommended in very-high-risk patients with FH if the treatment goals are not reached on maximal doses of statins and ezetimibe therapy or in patients who cannot tolerate statins [7,87,88,89,90]. A report by the American College of Cardiology (ACC) from 2022 recommends that if an additional reduction in the LDL-C levels is needed after the administration of the therapies mentioned above, the addition of inclisiran or bempedoic acid should be considered [91]. Moreover, patients with an inadequate response to lipid-lowering therapies can be considered candidates for lipid apheresis under the care of a lipid specialist [91].

The ESC and the EAS categorize HF patients without ASCVD or other major risk factors as high risk and recommend a ≥50% reduction in LDL-C from baseline and an LDL-C < 1.8 mmoL/L (<70 mg/dL) [7]. In FH patients at very high risk of ASCVD due to a prior history of ASCVD or another major risk factor, the LDL-C goals are a ≥50% reduction in LDL-C from baseline and an LDL-C < 1.4 mmoL/L (<55 mg/dL) [7]. The same goals are stated in the ESC guidelines on cardiovascular disease prevention from 2021 [87]. Recommendations for the treatment of patients with heterozygous familial hypercholesterolemia are shown in Table 4.

Additionally, treatment for patients with HoFH, the rarer and more dangerous variant of FH, should consist of aggressive lipid-lowering pharmacological therapy and, if available and necessary, lipoprotein apheresis [7]. It is recommended to maintain a maximally tolerated pharmacological therapy [7,53]. In the most severe cases of HoFH, liver transplantation can be considered, as it permanently corrects the molecular defect underlying the disease in the main organ involved in LDL clearance [17,53]. Recommendations for the treatment of patients with HoFH are shown in Table 5.

Early diagnosis and immediate treatment hold particular importance in the population of children, especially those with HoFH, who may develop cardiovascular disease very early in their lives if left untreated [17,92,93,94,95]. Data suggest that administering lipid-lowering therapy at an early age can decrease the LDL-C burden, improve the functions of the endothelium, slow down the progress of atherosclerosis, and thus, improve the coronary outcomes [7,96,97,98]. The ESC/EAS guidelines recommend that the treatment of children with FH includes a healthy lifestyle with a heart-healthy diet and statin treatment. Administering statins should be considered at 6–10 years of age [7]. Statin therapy should be initiated with low doses and then eventually increased to reach target values [99]. The ESC and the EAS recommend that the goal in children > 10 years of age is an LDL-C < 3.5 mmoL/L (<135 mg/dL), and at younger ages, a ≥50% reduction in LDL-C [7].

## 4. Pharmacological Therapy

### 4.1. Statins

Statins, otherwise known as 3-hydroxy-3methyl-glutaryl-CoA (HMG-CoA) reductase inhibitors, competitively inhibit HMG-CoA reductase and, thus, decrease the production of cholesterol in the liver, increase the expression of LDL receptors and promote the increased uptake of LDL from the blood [7,100]. The main effect of statins is a reduction in LDL-C; however, they also increase the levels of blood high-density lipoprotein cholesterol (HDL-C) [101].

The ESC/EAS guidelines for the management of dyslipidemias from 2019, the ESC guidelines on cardiovascular disease prevention from 2021, the multisociety guideline on the management of blood cholesterol from 2018 as well as the ACC report from 2022 point to statin therapy as a first-choice treatment option for FH [7,87,88,91]. However, statin monotherapy is often unsuccessful in reducing LDL-C to the target goals, and thus, the ESC/EAS recommends that in most patients, treatment should be initiated with high-intensity statin therapy in combination with ezetimibe [7,17].

High-intensity statins are shown to be capable of lowering the LDL-C levels by around 50% to 60% [102,103]. Statin therapy of moderate or low intensity, which is administered in patients who do not tolerate the high-dose, high-intensity treatment, usually lowers the LDL-C by 30–50% and by around 30%, respectively [103]. Statins decrease the incidence of ischemic heart disease (IHD) and the risk of complications of CVD, such as myocardial infarction (MI) or CAD death [85,104]. An observational study by Versmissen et al. [105] has found statin therapy to reduce the risk of cardiovascular events in patients with FH by 76%. Because of statins’ mechanism of action, homozygous FH individuals with null mutations on the *LDLR* gene are expected to be unresponsive to statin treatment, as these medications lower the LDL-C concentration to some extent by enhancing LDLR expression in the liver [17]. However, multiple studies have shown that these patients do respond to these drugs because of their alternative mechanisms of action, such as the reduction of very-low-density lipoprotein (VLDL) synthesis [106,107,108,109]. Yet, the effects of the treatment are lesser compared with those in FH patients [106].

Some of the adverse effects of statins include myopathy, which is the most clinically relevant, statin-associated muscle symptoms (SAMS) occurs in 10–15% of patients, while the most severe muscle damage, which is rhabdomyolysis, occurs in 1–3 patients for every 100,000 individuals per year [7,110,111,112,113]. Other side effects are a mild elevation of the ALT levels (in 0.5–2.0% of patients) and an increased risk of new-onset diabetes mellitus (by approximately 9%) [7,114]. Moreover, the administration of statin therapy in prepubescent children is considered controversial by some since statins possess the potential to inhibit the natural synthesis of steroid hormones [100,115]. However, several studies have shown that statins cause no severe adverse effects regarding growth, sexual development, and myo- or hepatotoxicity [98,116,117].

### 4.2. Ezetimibe and Statin–Ezetimibe Combination Therapy

Ezetimibe is an inhibitor of the Niemann-Pick C1-like 1 (NPC1L1) transporter and, thus, hampers the intestinal uptake of dietary and biliary cholesterol and reduces the transport of cholesterol to the liver, which responds by upregulating the expression of LDLR, which results in reduced LDL-C levels in the blood [7]. Since ezetimibe does not directly act via the expression of the LDL receptors, it is especially useful in treating patients with HoFH [100].

Ezetimibe has been assessed as a safe and effective drug. A study by Pearson et al. [118] has found that it decreased the LDL-C levels by approximately 18%, and another study on children with hypercholesterolemia by Clauss et al. [119] has shown similar results. The IMPROVE-IT trial by Cannon et al. [120] has revealed that adding ezetimibe to statins could reduce the LDL-C levels by an additional 20% and improve the cardiovascular outcomes even further. These findings were confirmed by several other studies, such as Gagné et al. [121] and van der Graaf et al. [122]. Moreover, the prespecified analysis of the ODYSSEY OUTCOMES randomized controlled trial by Ray et al. [123] has found that in patients on combination therapy of the highest tolerated dose of statin and either alirocumab or ezetimibe, for every additional 39 mg/dL lower achieved LDL-C level, the risk of major adverse cardiovascular events (MACE) was lowered by an additional 24%.

The current ESC/EAS guidelines and the ESC guidelines from 2021, the multisociety guideline on the management of blood cholesterol from 2018 as well as the ACC report from 2022 recommend the addition of ezetimibe to statin therapy as a first choice if the target LDL-C values are not achieved on station therapy alone [7,87,88,91]. However, some data show that most FH patients will still require additional therapies to reach their recommended goal [4].

The combination therapy of ezetimibe and statins has not been found to increase the occurrence of elevated creatine kinase (CK) levels beyond what is caused by statin treatment alone [124]. The incidence of severe liver failure with ezetimibe in monotherapy or in combination with statins is extremely rare [7]. The SEAS trial by Rossebø et al. [125] has found a small increase in the prevalence of cancer in patients treated with ezetimibe, which poses a concern for patients in need of lifelong therapy. However, additional data are needed on this topic. While the US Food and Drug Administration (FDA) has approved the administration of ezetimibe in children older than 10, the latest guidelines do not recommend the standard use of this medication in the pediatric population [7,100].

### 4.3. PCSK9 Inhibitors

Proprotein convertase subtilisin/kexin type 9 inhibitors promote the degradation of the PCSK9 enzyme and, thus, increase the expression of LDL receptors and intensify the removal of LDL-C from the blood [126].

As of now, the only PCSK9 inhibitors in common use are the human monoclonal antibodies (mAbs) alirocumab and evolocumab [7]. However, there are more agents under investigation, such as antisense nucleotide-based therapy and siRNAs (small interfering RNAs), which also act by inhibiting the PCSK9 protein and will be mentioned further below [127,128].

Clinical trials have shown that alirocumab and evolocumab, either administered in monotherapy or combined with statins and/or other lipid-lowering therapies, were able to decrease the LDL-C levels by circa 60%, depending on the dose, with the effectiveness of the treatment being mostly independent of any additional treatments [7]. Since statins raise the serum levels of the circulating PCSK9, the best effect of these mAbs has been shown in combination with statin therapy [7,129]. The ODYSSEY FH I, ODYSSEY FH II [130] and ODYSSEY HIGH FH [131] trials have all found that alirocumab was a well-tolerated drug that, in combination with statin therapy, allowed for a significant additional reduction in the LDL-C levels as well as a greater achievement of the LDL-C target levels in patients with FH. The RUTHERFORD [132] and the RUTHERFORD 2 [133] trials have both found that evolocumab exerted the same effect. The ODYSSEY ALTERNATIVE trial [134], which compared alirocumab with ezetimibe, has found that alirocumab reduced LDL-C to a greater extent in statin-intolerant patients compared to ezetimibe. The same results have been found for evolocumab in the GAUSS-3 trial [135].

The current ESC/EAS guidelines and the ESC guidelines from 2021, the multisociety guideline on the management of blood cholesterol from 2018 as well as the ACC report from 2022 recommend PCSK9 inhibitors for very-high-risk FH patients if their treatment goal is not reached on the maximally tolerated statins plus ezetimibe, as well as in FH patients who do not tolerate statins [7,87,88,89,91]. According to the ACC report from 2022, a preferred PCSK9 inhibitor should be a PCSK9 mAb because of the assessed efficacy, safety profile, and cardiovascular benefits presented in several clinical trials [91].

However, the response to the PCSK9 inhibitors is dependent on the degree of residual LDL receptor activity [92,136]. Therefore, it is only beneficial in patients with HeFH and the non-null phenotype HoFH [100]. It is advised that if a patient shows a >15% additional LDL-C reduction, PCSK9 therapy should be maintained, but if the response is lower, stopping this treatment should be considered [92].

The most common adverse effects of the PCSK9 inhibitors include itching at the site of injection and symptoms of the flu [7,137]. However, the possible issue of a long-term therapy with antibodies is the development of autoantibodies, and while very few cases of such a phenomenon have been reported, the matter should be closely observed [7]. Yet, as of now, open-label studies have assessed PCSK9 inhibitors to be effective and safe in long-term treatment (>3 years) [138].

### 4.4. Inclisiran

Inclisiran is a small interfering RNA that acts by selectively inhibiting the translation of the *PCSK9* gene and thus PCSK9 synthesis [139]. The ORION-9 trial [140] has assessed the effectiveness of inclisiran in combination with statins and with or without ezetimibe in patients with FH and found that it had an acceptable safety profile and was able to reduce LDL-C by an additional 48%. However, the efficacy of inclisiran, as well as other PCSK9-directed therapies, is LDLR-dependent and requires at least one normal allele to be effective [141]. Currently, the HPS4/TIMI65/ORION4 trial is studying the effects of inclisiran in patients with a prior MI or stroke, and its results will provide data on the long-term use of this medication [7,139]. A large, randomized placebo-controlled trial VICTORION-2P is also underway, which aims to assess the effect of the described drug on patients with ASCVD [142,143]. According to the ACC report from 2022, if inclisiran is to be administered, it should be used instead of PCSK9 mAb, as there is no evidence of additional benefits to support combining these agents [91].

### 4.5. VERVE-101

VERVE-101 is an investigational CRISPR base-editing agent that alters the DNA of the *PCSK9* gene and thus permanently inhibits the hepatic synthesis of the PCSK9 protein and therefore reduces the LDL-C levels in the blood [144]. A study on mice has shown that VERVE-101 was well tolerated and effectively lowered the LDL-C levels [144]. As of now, a phase 1b clinical trial, VT-1001, is evaluating the safety and pharmacodynamic profile of VERVE-101 in humans with HeFH; however, the results have not yet been posted [145].

### 4.6. Bempedoic Acid

Bempedoic acid is a cytosolic enzyme upstream of 3-hydroxy-3-methylglutaryl-coenzyme A reductase, which acts by reducing the synthesis of cholesterol by inhibiting the ATP citrate lyase [7]. Thus, it acts similarly to statins by decreasing the synthesis of cholesterol inside the cells and causing the upregulation of LDLR [141]. The CLEAR Harmony [146] and Wisdom [147] trials have assessed bempedoic acid’s safety and efficacy and have found that it was able to reduce the LDL-C levels by approximately 22.3% in patients with HeFH. This again proved that treatment strategies targeting the LDLR can be beneficial for patients with one healthy allele; however, the efficacy of bempedoic acid has not yet been studied in individuals with homozygous FH [141]. The CLEAR Outcomes trial [148], which assessed the efficacy of bempedoic acid in statin-intolerant patients, has found that treatment with bempedoic acid significantly lowered LDL-C and was correlated with a lower risk of major adverse cardiovascular events. However, the place of bempedoic acid is controversial in standard practice, as it is not yet recommended by the current ESC/EAS guidelines, while the ACC report from 2022 advises considering its administration if patients do not reach the LDL-C goals on statin treatment in combination with ezetimibe and PCSK9 inhibitors [91].

### 4.7. Bile Acid Sequestrants

Bile acid sequestrants bind the bile acids and prevent the reabsorption of cholesterol into the blood. Thus, they increase the metabolism of cholesterol into bile acids and intensify the synthesis of LDLR, which reduces the levels of LDL in the blood [7]. These drugs were frequently used in children with FH, since they are not systemically absorbed [100]. Cholestyramine and colestipol, the bile acid sequestrants of the older generation, are not used as often anymore, because of their lower effectiveness (they reduce the LDL levels only by approximately 10–20%) and the likelihood of causing dyspepsia [100]. Lately, a trail by Stein et al. [149] has shown that a new drug from this class, colesevelam, was able to significantly lower the LDL levels in children with HeFH. Colesevelam has also been reported to exert a beneficial effect on the glucose profile of hyperglycemic patients [150]. These revelations caused a renewed interest in this class of medication; however, it is not commonly used in standard practice [7,87].

### 4.8. Nicotinic Acid (Vitamin B3)

Nicotinic acid acts by blocking diacylglycerol acyltransferase-2 in the liver, which decreases the secretion of VLDL particles as well as IDL-C and LDL-C [151]. Moreover, it increases HDL-C and ApoA1 by promoting the synthesis of ApoA1 in the liver [151]. Studies have shown that niacin exerts no beneficial effect and has a poor tolerability profile [152,153]. As of now, no medication containing niacin is available in Europe, and nicotinic acid is not recommended in standard treatment [7].

### 4.9. Fibrates

Fibrates, which are agonists of the peroxisome proliferator-activated receptor-α (PPAR-α), regulate transcription factors in lipid and lipoprotein metabolism and thus the lower fasting triglycerides (TGs) levels and post-prandial TGs and TGs-rich lipoprotein (TRL) remnant particles [7,85]. It is estimated that fibrates are able to reduce the TGs levels by up to 50%, the LDL-C levels by ≤20% and increase the HDL-C levels by ≤20% [154]. A recent study, the PROMINENT trail [155], has evaluated the efficacy of pemafibrate, which is a selective peroxisome proliferator–activated receptor α (PPARα) modulator, in diabetic patients with hypertriglyceridemia and low HDL-C levels and has found that while pemafibrate was able to significantly lower triglyceride and VLDL and remnant cholesterol, the occurrence of cardiovascular events in these patients was not modified. Since data have shown that the overall CVD benefits of fibrates are inconclusive and questionable, and further confirmation is needed, therefore, these drugs are recommended as a part of the standard treatment [7].

### 4.10. Cholesteryl Ester Transfer Protein Inhibitors

Cholesteryl ester transfer protein (CEPT) promotes the conversion of HDL into LDL and VLDL, as well as the exchange of TGs from VLDL to LDL and HDL, and thus, decreases the HDL-C and increases the LDL-C levels, which results in advancing ASCVD and atherosclerosis [141,156]. Therefore, CEPT inhibitors like torcetrapib, dalcetrapib, anacetrapib and obicetrapib should stop this process and be effective in ASCVD prevention; however, studies have shown inconclusive data [141]. A study by Kastelein et al. [157] has assessed the efficacy of anacetrapib in combination with statin therapy in patients with HeFH and found that it reduced LDL-C by nearly 40%. However, the study was stopped due to concerns about anacetrapib accumulating in adipose tissue [141]. Another CEPTi, obicetrapib, has been shown to be able to reduce LDL-C up to 45% without accumulating in tissues [158]. Overall, CEPTi remains a potential approach to achieving LDL-C reduction in FH; however, as of now, CEPTi are not recommended in standard practice [7,43].

### 4.11. Evinacumab

Evinacumab is a mAb that binds to angiopoietin-like glycoprotein 3 (ANGPTL3) [141]. The binding of evinacumab to ANGPTL3 causes its inhibition, which in turn increases the activity of lipoprotein lipase (LPL) and endothelial lipase (EL), causing VLDL to be processed and removed before LDL particles are produced. As a result of the action of these enzymes, there is a significant reduction in the concentration of LDL-C, triglycerides and other lipoproteins in the circulation [159,160,161,162]. A study by Raal et al. [163] evaluated the effectiveness of evinacumab in patients with HoFH and found that it was able to reduce the LDL-C levels by 49% regardless of the different primary lipid-lowering treatments. Additionally, no difference in efficacy was observed in patients with null/null LDLR variants compared to those with non-null variants, and patients with less than 2% residual LDLR function achieved a 72% reduction in LDL-C compared to the placebo [141,163]. Overall, although evinacumab is an effective treatment option, it is not currently recommended as standard treatment [7].

### 4.12. Mipomersen

Mipomersen is an antisense oligonucleotide that binds the mRNA of ApoB-100 and promotes its degradation, thus reducing the synthesis of atherogenic lipids and lipoproteins such as LDL and Lp(a) [7,164]. Mipomersen can be used as an adjunct to lipid-lowering therapy and has been assessed to exert a dose-dependent reduction in the plasma levels of LDL-C, ApoB 100, Lp (a), and TGs in patients with HoFH [7,165]. However, concerns have been raised about the safety of this drug, as it has been noted that its use may promote the development of fatty liver disease. The mechanism that leads to this disease is related to the inhibition of the synthesis of apolipoprotein B (apoB), which probably contributes to the reduced production of very low density lipoproteins (VLDL), consequently causing the accumulation of triglycerides in the liver. In one of the studies, as part of the clinical development of mipomersen, seven patients underwent liver biopsy after using this drug (for 23–159 weeks) [166]. Histopathological results revealed that the patients had steatosis, without signs of significant inflammation or fibrosis. It should be emphasized that the described study had significant limitations, including a small study group, relatively short exposure time to the drug, and lack of baseline liver biopsies before drug administration. For the above reasons, mipomersen therapy is not currently recommended as standard treatment and further studies are needed to evaluate this drug for safety [7].

### 4.13. Lomitapide

Lomitapide is an inhibitor of microsomal triglyceride transport protein (MTP), which acts by reducing the synthesis and secretion of ApoB-containing lipoproteins in the liver and intestine and thus lowering the plasma LDL-C [85]. A study by Cuchel et al. [167], which assessed the efficacy of lomitapide in patients with HoFH, has found that it was able to reduce LDL-C by 50% and decrease the needed frequency of apheresis. However, the effect of lomitapide on the CV outcomes has not yet been determined, and the drug has been shown to increase the aminotransferase levels [7,167]. Currently, lomitapide is not yet recommended as a standard treatment [7].

### 4.14. Resmetirom

Resmetirom is a thyroid hormone receptor-beta-selective agonist that has recently been studied in patients with HeFH in a phase-2 double-blind, placebo-controlled, randomized trial [168]. It has been found to be able to significantly the lower LDL-C and other atherogenic lipid or lipoprotein levels and seems to be well tolerated as an adjunctive therapy as well [168]. However, as more studies are needed, resmetirom is not yet recommended as a standard treatment [7,69].

### 4.15. Gemcabene

Gemcabene, which is a lipid-modulating agent independent of the LDLR, acts by increasing the clearance of very-low-density lipoprotein from plasma, inhibiting cholesterol and triglyceride production in the liver and thus reducing the levels of very-low-density lipoprotein-C, LDL-C, Apo B, triglycerides, and high-sensitivity C-reactive protein [103]. A study by Gaudet et al. [169], which assessed the effectiveness of gemcabene in patients with HoFH as an adjunctive therapy to lipid-lowering treatment, has found that it was able to significantly reduce LDL-C. However, as more studies are needed, gemcabene is not yet recommended as a standard treatment [7].

In conclusion, when it comes to treating FH, a diverse range of medications do find applications. Nonetheless, not all drug groups are regarded as having equal standing. Certain ones, such as statins, ezetimibe, selected PCSK9 inhibitors, bile acid sequestrants and fibrates, have gained approval for use as a components of standard treatment. On the other hand, drugs like nicotinic acid, CEPT inhibitors, inclisiran, bempedoic acid, evinacumab, mipomersen, lomitapide, resmetirom and gemcabene are not recommended for common use at this moment [7]. What is interesting, even with combined therapy of the maximum dose statin plus ezetimibe and PCSK9 inhibitor, it is not always possible to achieve the desired LDL-C level in the blood, as per the 2019 ESC/EAS guidelines. There is a study indicating that out of 39 individuals with genetically confirmed FH, only 21 of them (53.84%) managed to achieve the target LDL-C concentration in line with the mentioned guidelines. However, none of the patients (0%) succeeded in reaching normal LDL-C values with oral combination therapy of statin plus ezetimibe or statin alone [170].

## 5. Non-Pharmacological Treatment

### 5.1. Diet

An appropriate diet in patients with familial hypercholesterolemia is very important, but it is not a key element of therapy [171]. In the case of patients who cannot start or do not tolerate lipid-lowering treatment, proper eating habits are necessary [172]. According to the recommendations of the ESC and the EAS, children should be introduced to a heart-healthy diet from an early age and, in turn, pharmacological treatment with statins should be contemplated at the age of 6–10 years [7]. It is very important that children remain under the supervision of an experienced dietitian who will introduce an appropriate nutritional strategy, ensuring the appropriate energy needs of the child as well as monitoring the growth curve [1]. It should be borne in mind that familial hypercholesterolemia is affiliated with significantly greater risk of premature atherosclerosis and cardiovascular diseases. An integral element in preventing these deadly diseases is a proper diet [2]. In addition to their direct impact on the development of CVD, dietary factors additionally modify the traditional risk factors, including blood pressure, glucose level, obesity, oxidative stress, inflammation and endothelial dysfunction [2,171,173]. Moreover, research shows that high consumption of saturated fat causes increased LDL-C levels, which in turn contributes to the occurrence of ASCVD [7]. Epidemiological studies show that it is beneficial to consume low-starch vegetables, fruits, nuts, legumes, fish, vegetable oils, and whole grain products, while the consumption of processed meat, salt and products containing refined simple sugars should be limited [7]. Dietary fiber also deserves special attention, as it has a beneficial effect on the entire digestive system and reduces cholesterol and glucose in the blood [174]. A higher intake of dietary fiber makes it a valuable tool in preventing and reducing the risk of atherosclerosis and cardiovascular diseases [175,176]. There are two basic types of fiber: soluble and insoluble. The main sources of soluble fiber are vegetables (carrots, broccoli, onions and artichokes), fruits, legumes, oats and barley, while insoluble fiber can be found in cereals and whole grain products [175,177].

### 5.2. Lipoprotein Apheresis

Another treatment for FH is lipoprotein apheresis. It is mainly used in children and adults with HoFH; however, it is crucial, especially in patients with severe HeFH, with very high LDL-C levels after maximally tolerated lipid-lowering therapy [4,17,103,178]. It is a safe and effective procedure that lowers blood cholesterol, reduces the likelihood of ASCVD and improves the long-term prognosis [179]. Studies have shown that this method can reduce the plasma LDL-C concentration by 50–75% [85,100]. Other benefits resulting from the use of lipoprotein apheresis include advancement of endothelial function, inhibition of the development or reduction of aortic valve stenosis, supravalvular stenosis of the aorta and coronary arteries [180,181]. Long-term treatment may reduce or eliminate yellow tufts [53,180]. Despite the many advantages of lipoprotein apheresis, it should not be forgotten that this method is quite difficult to access and, moreover, invasive and time-consuming, which may significantly affect the quality of life of patients [141].

### 5.3. Liver Transplant

Sometimes it may happen that the described therapies will be insufficient. Liver transplantation is a method that requires individual indications and is considered the last resort in the treatment of HoFH [182,183]. This procedure enables the replacement of dysfunctional hepatic LDL receptors in patients with HoFH, which significantly improves lipoprotein metabolism [184]. It is said that the concentration of LDL-C in the plasma can be reduced by as much as 80% [182,184,185]. Unfortunately, this method is not perfect. Due to the possibility of surgical complications, lack of donors, the need for lifelong immunosuppression, and the risk of severe immune reactions, this procedure is performed in exceptional cases [184,186].

### 5.4. Gene-Editing Technologies

One of the newest methods that may be used in the treatment of FH is CRISPR-Cas9 technology, which is currently still in the preclinical phase. In one study, the model was generated by mice with the nonsense point mutation LdlrE208X. The CRISPR-Cas 9 system was introduced into mouse somatic cells using an adenovirus (AAV), which resulted in the editing of the Ldlr gene affected by the mutation. The result of this process was the partial restoration of LDLR protein expression, which resulted in a decrease in LDL-C and a reduction in the area of atherosclerotic plaques. This is a very promising method, but further research is needed to accurately assess its safety and potential side effects [141,187,188].

## 6. Conclusions

Over the years, additional variants have been identified that have the potential to cause FH. It should be borne in mind that the large number of potential mutations causing FH, as well as the additional conceivable involvement of other genes and the likely presence of a polygenic background, mean that the FH phenotype may differ significantly in terms of the lipid profile and clinical symptoms, and therefore, it may respond differently to treatment. Importantly, the elevated LDL-C levels in FH are the main factor leading to the onset and development of atherosclerosis. This occurs because hypercholesterolemia is a factor that itself facilitates and intensifies the transport of LDL-C into the subendothelial space of the artery. Once it reaches the mentioned space, low-density lipoprotein cholesterol begins to trigger a cascade of events that ends with atheromatous plaque formation. Further progression of atherosclerosis results in the development of ASCVD, which may manifest in both acute and chronic ways. The most appropriate procedure in the case of newly diagnosed FH is lifestyle change and lipid-lowering treatment (statins in maximally tolerated doses, if necessary combined with ezetimibe and PCSK9 inhibitors). For patients with FH, the LDL-C targets have been strictly defined depending on the level of ASCVD risk. Unfortunately, in many cases, patients fail to achieve the set values, which significantly worsens their results. In addition to the pharmacological treatment of FH, non-pharmacological treatment is a very important form. Diet and building proper eating habits are one of the pillars of non-pharmacological treatment. Another form of non-pharmacological treatment is lipoprotein apheresis. Patients for whom traditional treatment methods do not provide the desired results may qualify for a liver transplant. This method is very rarely used and is associated with various complications after the procedure and the need to use immunosuppression for the rest of the patient’s life. One of the most modern methods that has recently been gaining importance in the potential treatment of FH is gene editing technology, which is in the preclinical phase.

## Figures and Tables

**Table 1:** Dutch Lipid Clinic Network criteria [9].

	Points
**Family history**	
First-degree relative with known premature coronary or vascular disease (men < 55 y, women < 60 y) or	1
First-degree relative with known LDL-C > 95th percentile	
First-degree relative with tendinous xanthomata and/or arcus cornealis or	2
Children aged < 18 y with LDL-C > 95th percentile	
**Clinical history**	
Patient with premature CAD (men < 55 y, women < 60 y)	2
Patient with premature cerebral or peripheral vascular disease (men < 55 y, women < 60 y)	1
**Physical examination**	
Tendon xanthomas	6
Corneal arcus < 45 y	4
**LDL-C levels**	
LDL-C >8.5 mmol/L (≥ 325 mg/dL)	8
LDL-C 6.58.4 mmol/L (251–325 mg/dL)	5
LDL-C 5.06.4 mmol/L (191–250 mg/dL)	3
LDL-C 4.04.9 mmol/L (155–190 mg/dL)	1
**DNA analysis**	
Functional mutation in the LDLR, apoB, or PCSK9 genes	8
**Diagnosis of FH**	
>8 points—a definite diagnosis	
6–8 points—a probable diagnosis	
3–5 points—a possible diagnosis	

Abbreviations: FH, familial hypercholesterolemia; y, years old; LDL-C, low-density lipoprotein cholesterol; CAD, coronary artery disease; APOB, gene encoding apolipoprotein B; LDLR, gene encoding the low-density lipoprotein receptor; PCSK9, gene encoding proprotein convertase subtilisin/kexin type 9 protein.

**Table 2:** Diagnostic criteria for HoFH [4].

**Clinical Criteria**
Untreated LDL-C > 10 mmol/L (>∼400 mg/dL) is suggestive of HoFH, requiring further investigation to confirm the diagnosis
Cutaneous or tendon xanthomas < 10 y and/or untreated elevated LDL-C levels in both parents with heterozygous FH
**Genetic Criteria**
Genetic confirmation of bi-allelic pathogenic/likely pathogenic variants on different chromosomes at the LDLR, APOB, PCSK9, or LDLRAP1 genes or ≥ 2 such variants at different loci

Abbreviations: FH, familial hypercholesterolemia; y, years old; HoFH, homozygous familial hypercholesterolemia; LDL-C, low-density lipoprotein cholesterol; APOB, gene encoding apolipoprotein B; LDLR, gene encoding the low-density lipoprotein receptor; LDLRAP1, gene encoding low-density lipoprotein receptor adaptor protein 1; PCSK9, gene encoding proprotein convertase subtilisin/kexin type 9 protein.

**Table 3:** Variants of the *LDLR* gene.

Class	Variants
I	Variants affecting receptor synthesis (e.g., FH French–Canadian-1, FH turkey, FH Nashville, FH Italy-1)
IIa	Variants causing post-translational defects likely to block transport of LDLR to the cell membrane completely (e.g., FH Saint Omer, FH Genoa, FH Naples, FH French–Canadian-2)
IIb	Variants causing post-translational defects that may block LDLR transport to the plasma membrane partially (e.g., FH Cape-Town-1, FH Mexico, FH Puerto Rico, FH Denver-2, FH Afrikaner-1)
III	Variants impaired in LDL binding (e.g., FH St. Louis, FH Paris-2, FH French–Canadian-4, FH London-2, FH-Leuven)
IV	Variants causing reduced ability to clathrin-coated pit-mediated endocytosis (e.g., FH Bahrain, FH Paris-3, FH Syria, FH Rochester, FH Osaka-1)
V	Variants causing abnormal recycling of LDLR (e.g., FH Osaka-2, FH Algeria, FH Afrikaner-2, FH Kuwait)

**Table 4:** Recommendations for the treatment of patients with heterozygous familial hypercholesterolemia by the ESC/EAS [7].

Recommendations	Class of Recommendation	Level of Evidence
It is recommended that FH patients with ASCVD or another major risk factor are treated as very high risk, and that those with no prior ASCVD or other risk factors are treated as high risk.	I	C
It is recommended that FH patients with ASCVD who are at a very high risk should be treated to reach a ≥50% reduction from baseline and an LDL-C < 1.4 mmoL/L (<55 mg/dL). If the target goals cannot be reached, administration of a drug combination is recommended.	I	C
An LDL-C reduction of ≥50% from baseline and an LDL-C target of < 1.4 mmoL/L (<55 mg/dL) should be considered as a primary prevention for FH patients at a very high risk.	IIa	C
Treatment with a PCSK9 inhibitor is recommended in very-high-risk patients with FH if the treatment goal is not reached on maximum tolerated doses of statin and ezetimibe combination.	I	C
Children with FH should follow a healthy diet and start statin therapy at 8–10 years old. The target levels for treatment should be LDL-C < 3.5 mmoL/L (<135 mg/dL) at >10 years of age.	IIa	C

Abbreviations: ASCVD—atherosclerotic cardiovascular disease; FH—familial hypercholesterolemia; LDL-C—low-density lipoprotein cholesterol; PCSK9—proprotein convertase subtilisin/kexin type 9. Classes of recommendation: Class I—evidence and/or general agreement that a given treatment or procedure is beneficial, useful, effective; Class IIa—weight of evidence/opinion is in favor of usefulness/efficacy. Levels of evidence: C—consensus of opinion of experts and/or small studies, retrospective studies, registries.

**Table 5:** Recommendations for treatment of patients with homozygous familial hypercholesterolemia by the European Atherosclerosis Society (EAS) [4].

Recommendations for Patients with HoFH
The LDL-C goal for adult individuals with HoFH is <1.8 mmol/L (<70 mg/dL), and <1.4 mmol/L (<55 mg/dL) for HoFH patients with additional risks, such as an elevated Lp(a) level or diabetes mellitus, or with an established ASCVD.
The LDL-C goal after administering lipid-lowering therapies in pediatric population without established ASCVD is < 3 mmol/L (<115 mg/dL). The target LDL-C level is lower in children diagnosed with ASCVD.
A combination of high-intensity statin and ezetimibe is recommended to be administered at diagnosis.
Within 8 weeks of such treatment, additional therapy with a PCSK9 inhibitor should be considered and added if available. It is suggested that such therapy should be maintained if an >15% additional LDL-C reduction is achieved; however, if patient’s response is poor, discontinuation should be considered.
If the LDL-C is still above the target level, lomitapide and/or ANGPTL3-directed therapy should be considered with or without lipoprotein apheresis.
If PCSK9-directed therapy or novel therapies such as lomitapide and/or ANGPTL3-directed therapies are not available or affordable, lipoprotein apheresis should be considered.

Abbreviations: ANGPTL3—angiopoietin-like 3; ASCVD—atherosclerotic cardiovascular disease; HoFH—homozygous familial hypercholesterolemia; LDL-C—low-density lipoprotein cholesterol; PCSK9, proprotein convertase subtilisin/kexin type 9.

## Data Availability

The data used in this article were sourced from materials mentioned in the References section.
